# CT Morphometric Analysis of the Medial Femoral Condyle in an Indian Population: A Comparative Study of Unicompartmental Knee Arthroplasty Implants

**DOI:** 10.7759/cureus.70454

**Published:** 2024-09-29

**Authors:** Kedar Ahuja, Ashraf Shaikh, Devansh Lalwani, Mohan M Desai

**Affiliations:** 1 Department of Orthopaedics, Shivneri Hospital, Ulhasnagar, IND; 2 Department of Orthopaedics, King Edward Memorial (KEM) Hospital and Seth Gordhandas Sunderdas Medical College (GSMC), Mumbai, IND; 3 Department of General Medicine, King Edward Memorial (KEM) Hospital and Seth Gordhandas Sunderdas Medical College (GSMC), Mumbai, IND

**Keywords:** ct morphometric analysis, implant size compatibility, indian population knee implants, medial femoral condyle, unicompartmental knee arthroplasty

## Abstract

Purpose

The objective of this study was to evaluate the compatibility of the medial femoral condyle (MFC) dimensions in the Indian population with the standard femoral components of three commercially available unicompartmental knee arthroplasty (UKA) implants.

Methods

This analytical study utilized CT morphometric data from 100 consecutive non-arthritic adult knees. MFC dimensions were measured to assess their compatibility with UKA prostheses. The proportion of knees that could be optimally replaced using three commercially available UKA femoral implant systems, Link, DePuy, and Smith & Nephew, was calculated.

Results

The study cohort comprised 56 males and 44 females, with a mean age of 43.28 ± 10.53 years. For the Link implant system, the 'small' size was the most compatible, fitting 43% (43) of the patients, followed by 'medium-small' (35%, n = 35) and 'medium' (16%, n = 16). For the DePuy Sigma Uni implant, the best fit was 'size 1' for 38% (38) of the patients, followed by 'size 3' (25%, n = 25), 'size 4' (13%, n = 13), 'size 2' (10%, n = 10), and 'size 5' (2%, n = 2). In the case of the Smith & Nephew system, 'size 2' was the best fit for 30% (30) of the knees, followed by 'size 3' (27%, n = 27), 'size 4' (15%, n = 15), 'size 1' (12%, n = 12), and 'size 5' (8%, n = 8). Overhang of the femoral component, due to the MFC being smaller than the smallest available femoral component size, was observed in 6% (6) of knees with Link implants, 12% (12) with DePuy implants, and 8% (8) with Smith & Nephew implants.

Conclusion

The findings indicate that the most suitable femoral component sizes for Indian knees were 'small' in the Link system (43%, n = 43), 'size 1' in the DePuy system (38%, n = 38), and 'size 2' in the Smith & Nephew system (30%, n = 30). The results suggest a need for implant manufacturers to consider developing customized components tailored to the Indian population to enhance fit and minimize the occurrence of femoral component overhang.

## Introduction

Osteoarthritis (OA) is a prevalent degenerative disorder affecting the knee joint, with the medial compartment of the tibiofemoral articulation being the most commonly impacted area [1,2]. Unicompartmental knee arthroplasty (UKA) has emerged as a highly effective treatment for unicompartmental knee arthritis, offering significant advantages such as preservation of the patient's native bone, minimized surgical trauma, and enhanced postoperative recovery [3]. However, the success of UKA is closely linked to the accurate sizing of the femoral component. An oversized femoral component can lead to anterior overstuffing, which in turn increases the risk of patellofemoral complications and soft-tissue impingement [4]. Therefore, achieving an optimal fit between the implant and the resected knee surface is crucial for a successful outcome in UKA procedures.

The majority of UKA implants currently available on the market are designed based on anthropometric data derived from Western populations [5]. However, significant racial differences exist between Indian and Western populations in terms of physical traits and dimensions, which may influence the suitability of these implants for Indian patients. Despite these differences, there is a paucity of research comparing the dimensions of UKA implants to the anatomical characteristics of the Indian population, particularly concerning the medial femoral condyle.

This study aims to address this gap by comparing the anteroposterior (AP) and mediolateral (ML) dimensions of the medial femoral condyle in the Indian population with the standard-sized femoral components of commercially available UKA implants. Additionally, the study seeks to identify the most commonly suitable femoral implant sizes for Indian knees, thereby providing valuable insights for optimizing UKA outcomes in this demographic.

## Materials and methods

Methods

This analytical, cross-sectional study was conducted at a tertiary care hospital following approval from the Institutional Ethics Committee (Project No. EC/223/2018). Sample size estimation and sampling were performed based on Cohen’s d (effect size) calculation [6], assuming a significance level of 5% (α) and a statistical power of 80% (1 − β); this resulted in a calculated sample size of 100 patients.

The study included skeletally mature patients (above 18 years) who underwent CT scans of normal knees, excluding those with any pathologies affecting femoral condyle morphometry, such as fractures, osteoarthritis, rheumatoid arthritis, growth abnormalities, or metabolic disorders. Skeletally immature individuals or those with abnormal knee findings were also excluded.

During the scans, patients were positioned supine, with knees relaxed and in extension. A 3D-CT scan (120 kVp, 50 mAs; Philips Healthcare) was performed to acquire CT slices with a thickness of 0.5 mm and a resolution of 512 × 512 pixels. Two surgeons measured the MFC dimensions using OsiriX DICOM (Digital Imaging and Communications in Medicine) viewer software for Mac OS.

Morphometric measurements of the medial femoral condyle (MFC)

**Figure 1:**
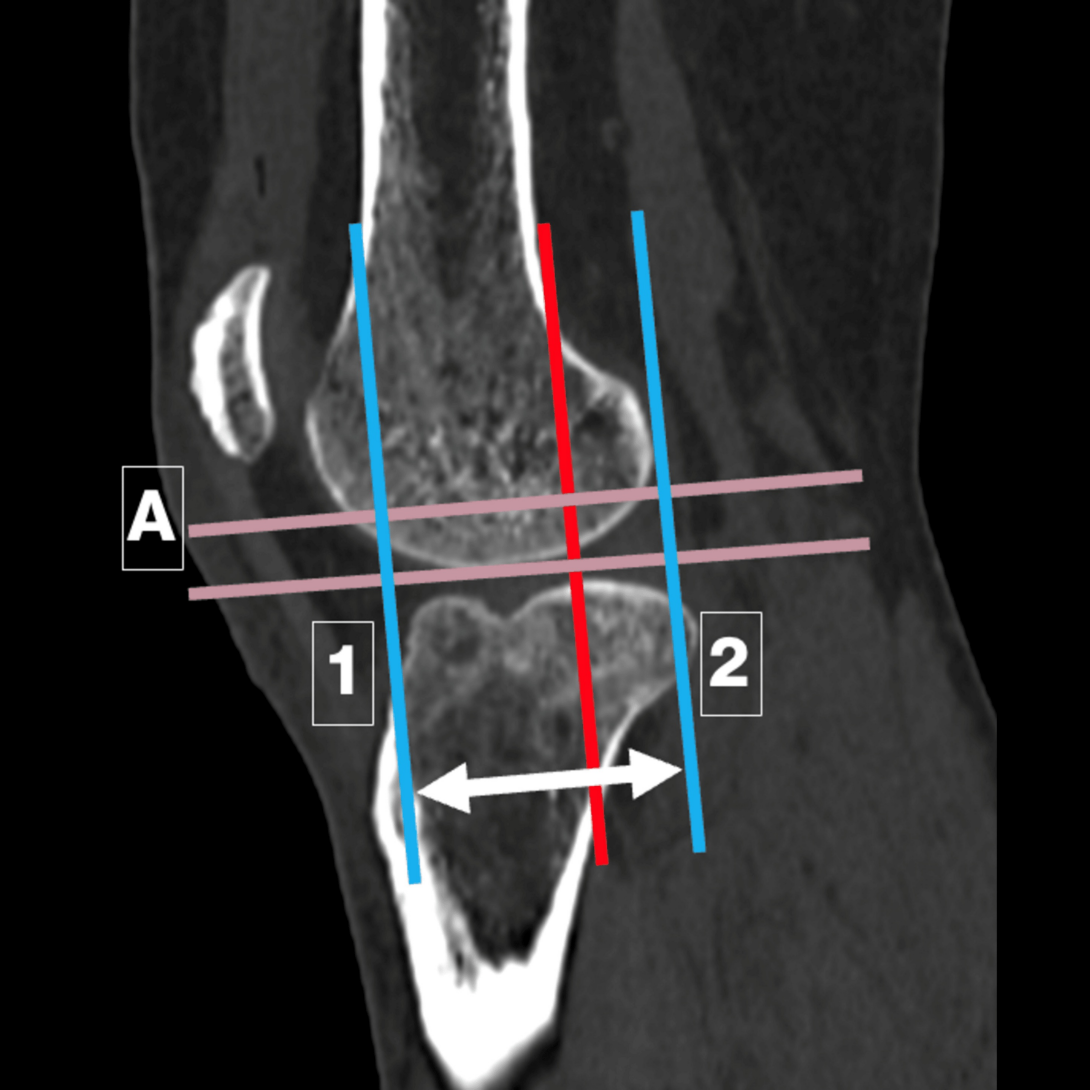
Sagittal CT image to measure the anteroposterior (AP) dimensions and compare them with implant sizes. Red line: drawn across the posterior femoral cortex. Grey line: drawn across the distal femoral cortex. Line A: line drawn 6.7 mm proximal and parallel to the grey line along the distal femoral cortex. Lines 1 and 2: two more lines transecting Line A at the anterior and posterior cortices, drawn parallel to each other at maximum AP dimensions.

**Figure 2:**
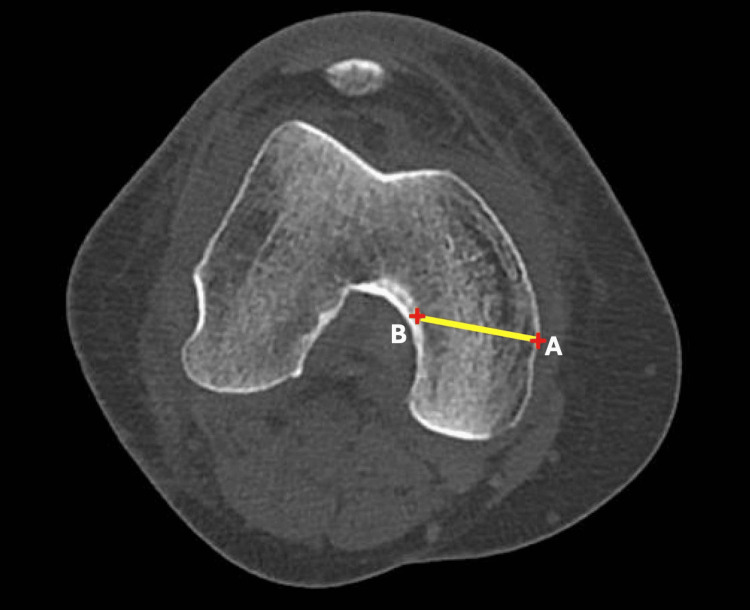
CT image of the distal femur showing mediolateral dimensions of the medial condyle at the distal cut. Line AB: mediolateral dimension of the medial condyle, measured at the level of the distal femoral cut.

A line was drawn along the posterior femoral cortex corresponding to the posterior peg of the UKA implant. The minimal resection was 6.7 mm distal to the distal femoral cortex, so a line perpendicular to the posterior femoral cortex was drawn 6.7 mm proximal to the distal femoral cortex (Line A). Two additional lines transecting Line A at the anterior and posterior cortices were drawn parallel to each other at the maximum anteroposterior (AP) dimensions (Lines 1 and 2) (Figure 1). The distance between Lines 1 and 2 was compared with the AP dimensions of different commercially available implant sizes. The mediolateral (ML) length was measured along the same axis (Line AB) in axial cuts. Overhang was defined as an AP fit greater than 2 mm beyond the femoral condyle (Figure 2).

To ensure measurement consistency, inter-observer reliability was assessed. Two independent observers measured the MFC dimensions (AP and ML lengths). If a discrepancy greater than 1 mm occurred between their measurements, a third observer resolved the disagreement. The inter-observer reliability was calculated using the intraclass correlation coefficient (ICC), with values greater than 0.75 indicating good reliability and greater than 0.90 indicating excellent reliability. The ICC was computed using a two-way mixed-effects model for absolute agreement.

Statistical analysis

Patient demographic data and the ML and AP measurements of the MFC were summarized as mean and SD (mean ± SD). The best-fit lines for each of the implant systems were determined using the least squares regression method. The dimensions of the patients’ MFCs were then compared with those of various commercially available implants, specifically Link, DePuy, and Smith & Nephew implants.

Statistical analysis was conducted using Microsoft Excel and SPSS version 20 (IBM Corp, Armonk, NY). A chi-square test of association was utilized to compare the medial femoral condylar parameters across the different UKA implants as well as to compare male and female patients. A p-value ≤0.05 was considered statistically significant.

## Results

There were 44 females and 56 males in this study, resulting in a male-to-female ratio of 1.27:1. The mean age of the study participants was 43.28 ± 10.53 years. The average ML diameter was 20.99 ± 1.44 mm, and the average AP diameter was 47.56 ± 3.55 mm. The minimum ML diameter was 17.3 mm, and the minimum AP diameter was 39.8 mm (Table 1).

**Table 1:** Descriptive statistics. AP: Anteroposterior; ML: Mediolateral.

Parameter	Minimum	Maximum	Mean	SD
Age	21	62	43.28	10.53
Patients AP	39.8	54.3	47.56	3.55
Patients ML	17.3	23.5	20.99	1.44

The inter-observer reliability for measuring the MFC dimensions (AP and ML lengths) was excellent. The intraclass correlation coefficient (ICC) for AP measurements was 0.91 (95% CI: 0.85-0.94), and for ML measurements, the ICC was 0.93 (95% CI: 0.88-0.96). Any discrepancies greater than 1 mm were resolved by a third observer.

The morphometric analysis revealed distinct trends in the best-fit sizes among the three commercially available UKA implant systems: Link, DePuy, and Smith & Nephew.

**Figure 3:**
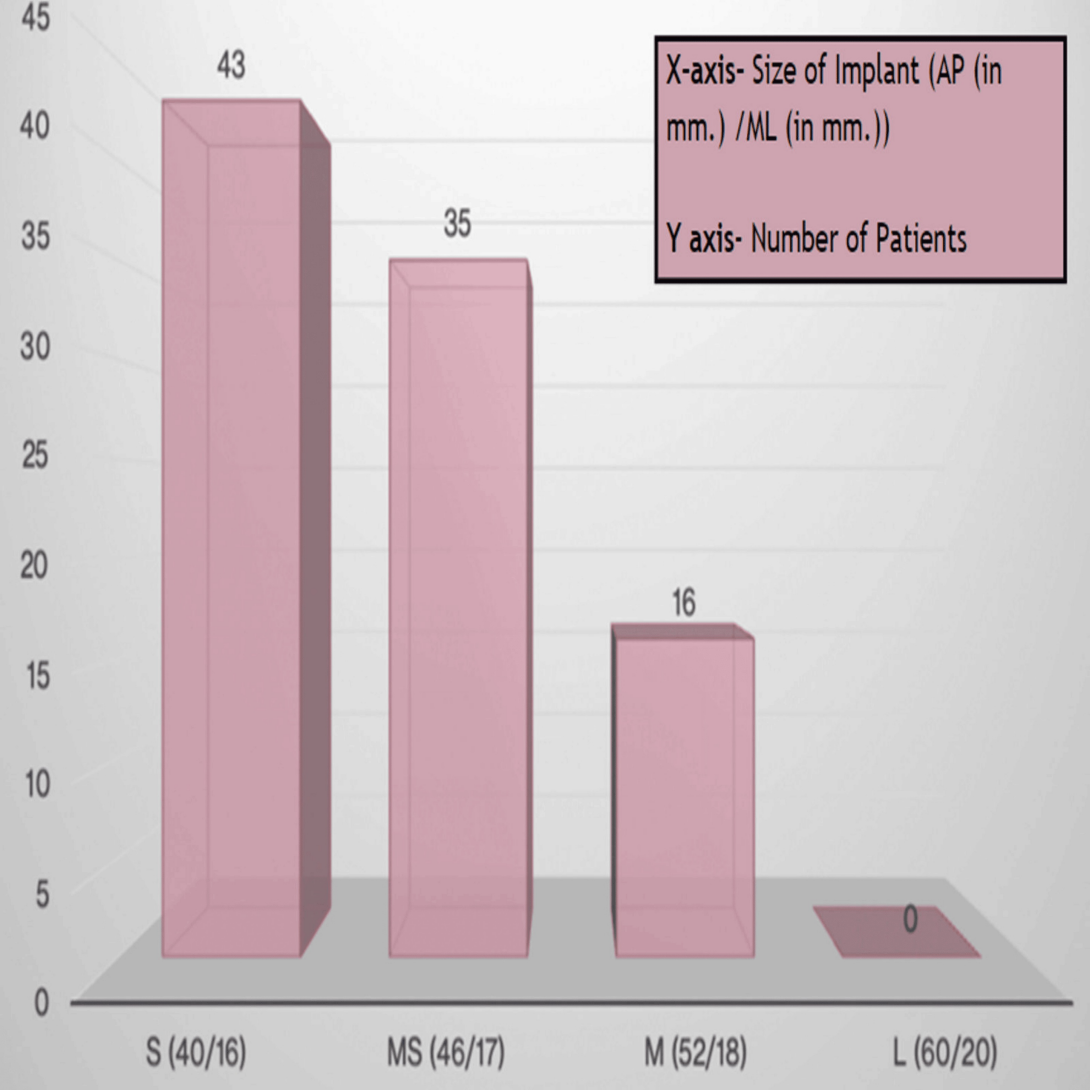
Link size best fit. S: Small; MS: Medium small; M: Medium; L: Large.

Among the Link implant systems, the 'small' size was the most suitable for 43% (43) of the patients, indicating a notable prevalence of this category. Similarly, the 'medium-small' size exhibited an optimal fit in 35% (35) of the patients, underscoring its relevance in a substantial proportion of the study cohort. The 'medium' size, although less common, still demonstrated a favorable fit in 16% (16) of patients (Figure 3).

**Figure 4:**
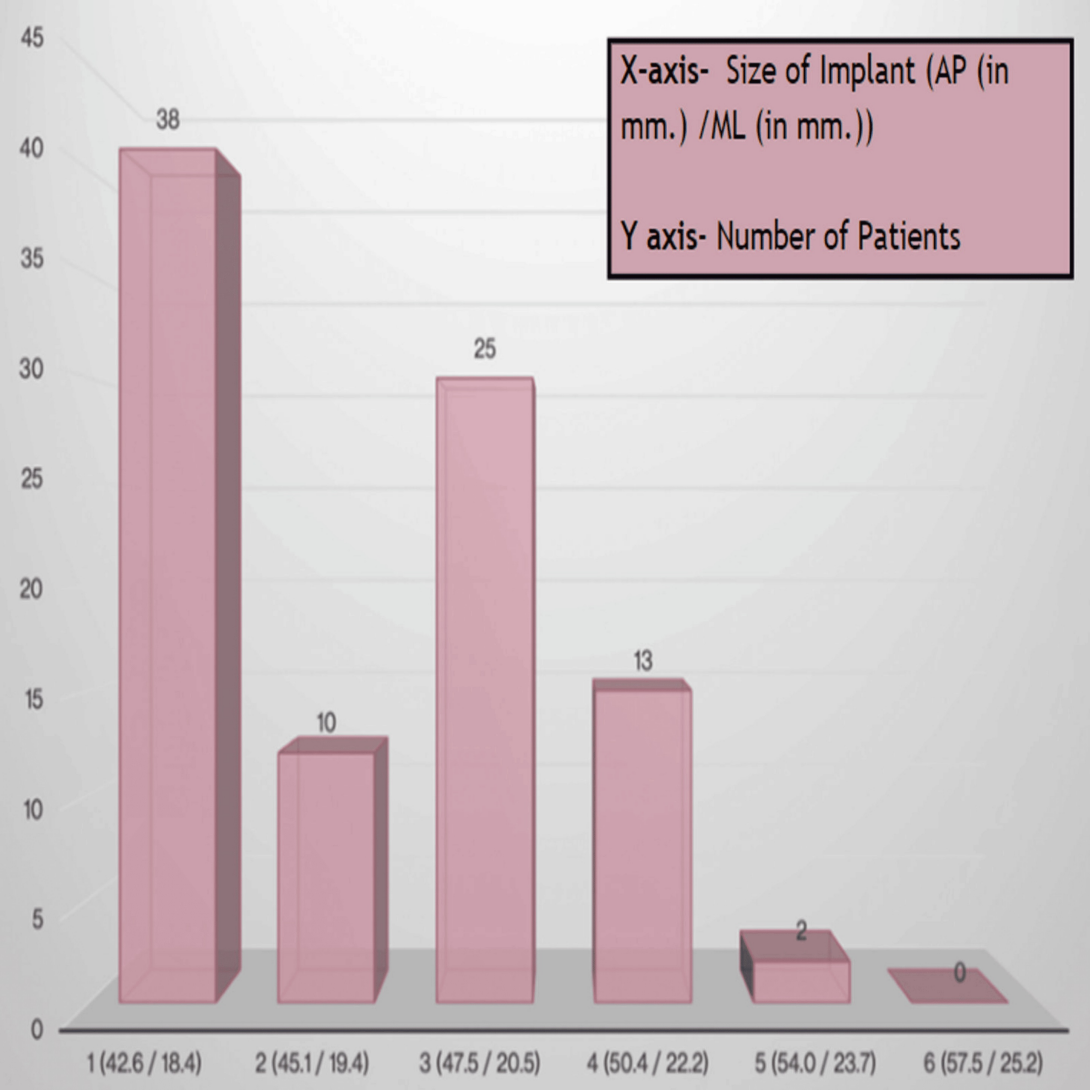
DePuy best fit.

Among the DePuy implants, size 1 was the best fit for 38% (38) of the patients, indicating its compatibility with a significant portion of the study population. Subsequent sizes demonstrated varying degrees of suitability, with size 3 indicating an optimal fit in 25% (25) of the patients. Sizes 4, 2, and 5 exhibited successively diminishing percentages of optimal fits (Figure 4).

**Figure 5:**
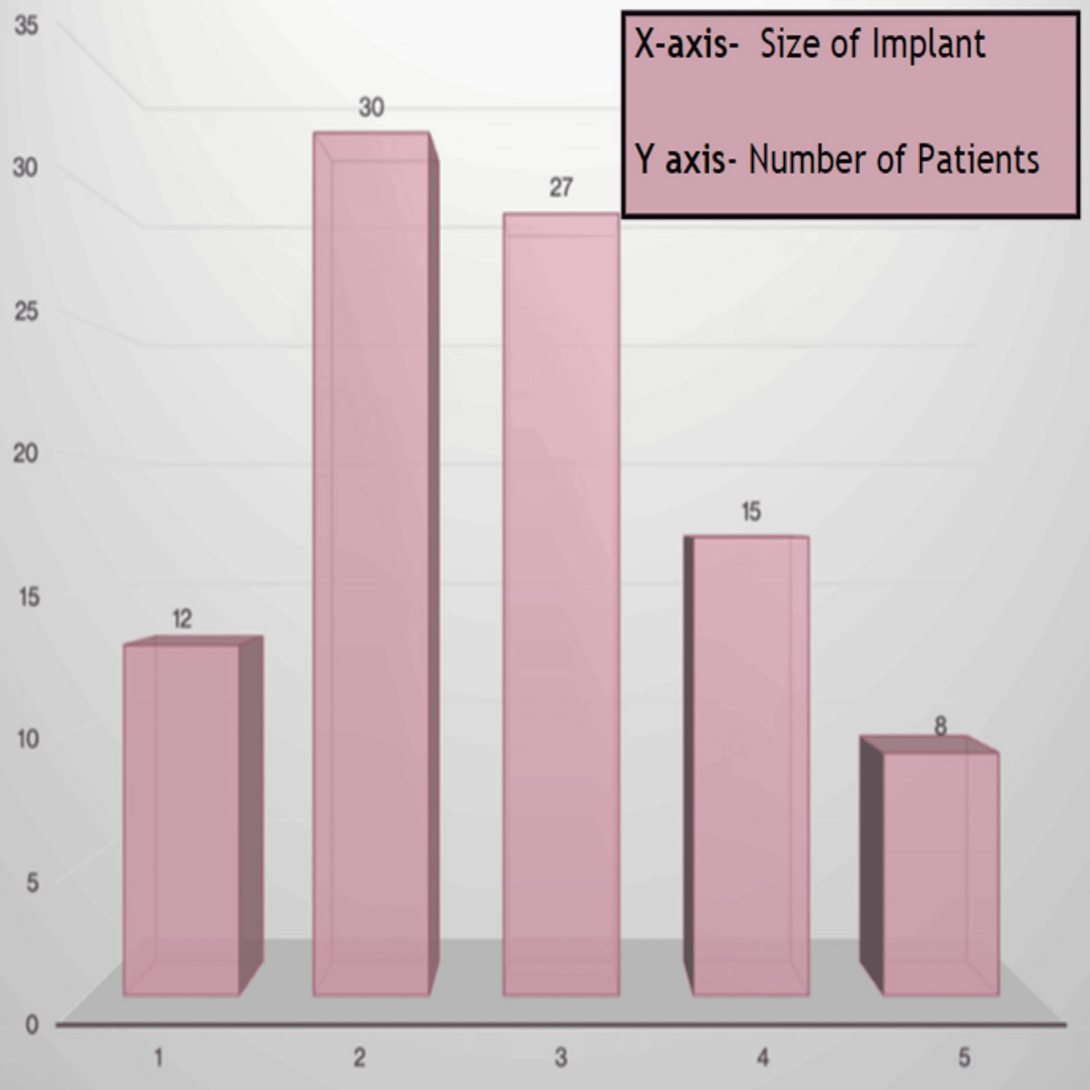
Smith & Nephew best fit.

Among the Smith & Nephew implant systems, size 2 emerged as the most fitting choice for 30% (30) of the patients, closely followed by size 3 at 27% (27). The subsequent sizes, 4, 1, and 5, showed descending percentages of optimal fits (Figure 5).

Despite the comparison with the smallest implants available, a notable occurrence of femoral overhang was observed across all three implant systems. Specifically, 6% (6) of patients with Link implants, 12% (12) with DePuy implants, and 8% (8) with Smith & Nephew implants exhibited femoral overhang.

## Discussion

Historical development and evolution of UKA

The field of knee arthroplasty has undergone significant advancements, particularly in implant design and surgical techniques. UKA has emerged as an effective treatment option, providing a less invasive alternative to total knee replacement by preserving healthy tissues and facilitating quicker postoperative recovery [5]. As the prevalence of OA, particularly in the medial compartment of the knee, has increased, the demand for effective, minimally invasive treatments has also grown. UKA was developed to meet this demand, offering a targeted approach for patients with unicompartmental knee arthritis.

The development of UKA dates back to the 1970s, with the work of Insall J and Ranawat C, who introduced partial knee replacement to address isolated compartmental degeneration [7]. A notable advancement occurred in 1993 with the introduction of the Oxford meniscal knee by Carr A et al., which demonstrated improved survival rates and functional outcomes [1]. Over time, UKA has evolved from early fixed-bearing designs to modern mobile-bearing systems, which have enhanced implant stability and longevity. Additionally, various implant systems have been introduced, each with specific features aimed at improving clinical outcomes [8,9].

Challenges in achieving optimal fit

Despite progress in UKA development, achieving an optimal fit between the implant components and the resected knee surface remains challenging [10]. This study aligns with previous research, including that by Hitt K et al. [11], which highlighted the difficulties in achieving complete coverage of the exposed cortical rim with current knee arthroplasty systems. Our findings indicate that even the most optimized implants cover only 76% of the cortical rim, underscoring the ongoing challenges in achieving a precise fit for knee implants and the importance of detailed considerations in implant design.

Our evaluation of three commercially available UKA implants revealed variations in overhang prevalence, with the lowest incidence observed in Link implants, followed by Smith & Nephew, and the highest in DePuy implants. The significant fit observed with the Link Sled 'small' size (43%, n=43) suggests a closer match to the MFC dimensions in the Indian population, indicating potential for better outcomes.

Femoral component overhang and its clinical implications

Femoral component overhang can lead to complications such as soft tissue irritation, pain, restricted movement, and increased wear on surrounding structures, which may reduce implant longevity and lead to revision surgeries [12]. Clinically, this can manifest as persistent knee pain and decreased postoperative satisfaction. Surgeons can mitigate these risks through meticulous preoperative planning, selecting appropriately sized implants that match patient anatomy, and utilizing intraoperative tools, such as trial components, to assess for overhang before final fixation. Additionally, advanced imaging techniques, like 3D templating, can be used to improve the accuracy of implant positioning. When overhang is detected intraoperatively, surgeons may opt for undersizing or using gender-specific implants to reduce the risk of soft tissue impingement and other complications [4].

Population-specific anatomical variations

This study highlights the importance of considering population-specific anatomical variations in implant design. Our findings are consistent with those of Cheng et al., who examined femoral implants in the Chinese population and reported overhangs in all AP dimensions [13]. This underscores the need to understand population-specific anatomical differences to improve implant design. Similarly, Fitz W et al.'s study on cadavers noted the mismatch between current UKA dimensions and measured sizes, particularly challenges in achieving an optimal fit, especially in males [14].

Gender-based anatomical differences further complicate femoral morphology considerations, as explored by Yan M et al. [15]. The statistically significant sex-based variation in the ML/AP ratios of the femoral condyles adds complexity to implant design. In our study, the incidence of overhangs was generally higher in females than in males across all three UKA implants, although the difference was not statistically significant.

Global perspectives on implant design

Studies on diverse populations, such as Kim JB et al.'s investigation of the femoral morphology of Korean women, highlight the inadequacy of implant designs based on data from Caucasian populations [16]. Similarly, Surendran S et al.'s research on the anthropometry of resected medial tibial condyles in Korean cadavers identified challenges in achieving an optimal fit, with specific concerns regarding ML overhang for certain tibial component designs [17]. The findings of Kantanavar R et al.'s study on the compatibility of medial tibial condyle morphometry in the Indian population with contemporary UKA prostheses align with our results [18].

Implications for clinical practice and future directions

This study adds to the growing body of evidence emphasizing the need for population-specific considerations in knee implant design [19, 20]. The observed anatomical variations across different populations present challenges in achieving a universally optimal fit [21]. These findings suggest that a one-size-fits-all approach to UKA implants may not be suitable, particularly in diverse populations such as India. The variations in optimal fit among different implants highlight the necessity for implant manufacturers to consider the specific anatomical characteristics of different populations. Customizing implants based on regional anatomical variations could lead to improved patient outcomes, reduced complications, and enhanced long-term success of UKA.

This study is limited by its single-center design, which may not reflect broader clinical practices, and by its focus on an Indian population, limiting generalizability to other ethnic groups. Additionally, the small sample size, the lack of long-term clinical outcome data, and the use of only three implant systems may not fully capture the diversity of available options in UKA. Moreover, the use of CT scans from non-arthritic knees, while useful for measuring baseline morphometric data, may not fully represent the changes in knee anatomy caused by OA or other degenerative conditions, which could affect implant fit and performance in real-world surgical settings. Future research should prioritize large-scale, multicenter studies that include diverse populations, along with comprehensive data on anatomical variations and implant performance. Collaborative efforts among researchers, surgeons, and implant manufacturers are essential to drive innovation in knee implant design, optimizing outcomes and improving the quality of life for patients undergoing UKA worldwide.

## Conclusions

Our study revealed that among the three commercially available implants compared, the best fit for Indian knees was observed with the ‘small’ implants in Link (43%), ‘size 1’ in DePuy implants (38%), and ‘size 2’ in Smith & Nephew implants (30%). Based on the findings of this study, we suggest thorough consideration of population measurements to ensure that the implants are the best fit for the majority of the Indian population. Since UKA is a long-term treatment modality and prognosis is mainly dependent on how well the implants fit the patient’s morphology, we recommend a customized approach for the Indian population in the planning of UKA components by commercial implant companies.
